# A novel non-invasive method to detect excessively high respiratory effort and dynamic transpulmonary driving pressure during mechanical ventilation

**DOI:** 10.1186/s13054-019-2617-0

**Published:** 2019-11-06

**Authors:** Michele Bertoni, Irene Telias, Martin Urner, Michael Long, Lorenzo Del Sorbo, Eddy Fan, Christer Sinderby, Jennifer Beck, Ling Liu, Haibo Qiu, Jenna Wong, Arthur S. Slutsky, Niall D. Ferguson, Laurent J. Brochard, Ewan C. Goligher

**Affiliations:** 1 Department of Anesthesia, Critical Care and Emergency, Spedali Civili di Brescia, University of Brescia, UNIBS, Brescia, Italy; 2 Department of Medical and Surgical Specialities, Radiological Sciences and Public Health, University of Brescia, UNIBS, Brescia, Italy; 3 Interdepartmental Division of Critical Care Medicine, University of Toronto, Toronto, Canada; 4 Keenan Centre for Biomedical Research, Li Ka Shing Knowledge Institute, St. Michael’s Hospital, Toronto, Canada; 5 Division of Respirology, Department of Medicine, University Health Network and University of Toronto, Toronto, Canada; 6 Respiratory Therapy, University Health Network, Toronto, Canada; 7 Institute for Health Policy, Management, and Evaluation, University of Toronto, Toronto, Canada; 8 Department of Critical Care Medicine, Zhongda Hospital, Southeast University, Nanjing, China; 9 Department of Physiology, University of Toronto, Toronto, Canada; 10 Toronto General Hospital Research Institute, Toronto, Canada; 11 Toronto General Hospital, 585 University Ave., Peter Munk Building, 11th Floor, Room 192, Toronto, ON M5G 2N2 Canada

**Keywords:** Mechanical ventilation, Artificial respiration, Acute lung injury, Myotrauma, Respiratory monitoring

## Abstract

**Background:**

Excessive respiratory muscle effort during mechanical ventilation may cause patient self-inflicted lung injury and load-induced diaphragm myotrauma, but there are no non-invasive methods to reliably detect elevated transpulmonary driving pressure and elevated respiratory muscle effort during assisted ventilation. We hypothesized that the swing in airway pressure generated by respiratory muscle effort under assisted ventilation when the airway is briefly occluded (Δ*P*_occ_) could be used as a highly feasible non-invasive technique to screen for these conditions.

**Methods:**

Respiratory muscle pressure (*P*_mus_), dynamic transpulmonary driving pressure (Δ*P*_L,dyn_, the difference between peak and end-expiratory transpulmonary pressure), and Δ*P*_occ_ were measured daily in mechanically ventilated patients in two ICUs in Toronto, Canada. A conversion factor to predict Δ*P*_L,dyn_ and *P*_mus_ from Δ*P*_occ_ was derived and validated using cross-validation. External validity was assessed in an independent cohort (Nanjing, China).

**Results:**

Fifty-two daily recordings were collected in 16 patients. In this sample, *P*_mus_ and Δ*P*_L_ were frequently excessively high: *P*_mus_ exceeded 10 cm H_2_O on 84% of study days and Δ*P*_L,dyn_ exceeded 15 cm H_2_O on 53% of study days. Δ*P*_occ_ measurements accurately detected *P*_mus_ > 10 cm H_2_O (AUROC 0.92, 95% CI 0.83–0.97) and Δ*P*_L,dyn_ > 15 cm H_2_O (AUROC 0.93, 95% CI 0.86–0.99). In the external validation cohort (*n* = 12), estimating *P*_mus_ and Δ*P*_L,dyn_ from Δ*P*_occ_ measurements detected excessively high *P*_mus_ and Δ*P*_L,dyn_ with similar accuracy (AUROC ≥ 0.94).

**Conclusions:**

Measuring Δ*P*_occ_ enables accurate non-invasive detection of elevated respiratory muscle pressure and transpulmonary driving pressure. Excessive respiratory effort and transpulmonary driving pressure may be frequent in spontaneously breathing ventilated patients.

## Introduction

Patient inspiratory effort during mechanical ventilation may have both beneficial and deleterious effects. Inspiratory effort increases tidal volume and global dynamic lung stress (quantified by transpulmonary driving pressure, Δ*P*_L_) in pressure-targeted modes of ventilation, potentially leading to lung injury. Vigorous inspiratory efforts can generate pendelluft and amplify regional lung stress and strain, causing regional lung injury even in volume-cycled modes of ventilation [1, 2]. The amplitude of this regional stress is reflected by the dynamic transpulmonary driving pressure, Δ*P*_L,dyn_ [3]. Excess diaphragmatic loading may impair systemic oxygen delivery and cause diaphragm muscle injury [4, 5]. The level of inspiratory effort during the first 3 days of ventilation was recently shown to predict the duration of ventilation and ICU admission [6]. Respiratory drive and effort are frequently elevated in patients with respiratory failure because of pain, anxiety, delirium, inadequate ventilatory assistance, and dyspnea [7, 8]. Therefore, patient inspiratory effort merits close attention during mechanical ventilation.

Inspiratory effort (quantified by respiratory muscle pressure, *P*_mus_) is not routinely monitored during mechanical ventilation. Although several monitoring techniques are available (e.g., esophageal manometry [9], diaphragm electrical activity (Edi) [10], diaphragm ultrasound [11]), they require appropriate equipment, proficiency, and time, making it difficult for busy clinicians to assess inspiratory effort as part of routine respiratory monitoring. *P*_0.1_ is a simple and widely available method for estimating respiratory drive during mechanical ventilation [12], but it provides little information about the magnitude of dynamic lung stress generated by the combined effects of the ventilator and patient respiratory effort. Plateau pressure and driving pressure are used to detect excess lung stress during controlled mechanical ventilation [13], but these measurements may not be reliable in the presence of inspiratory effort as they can underestimate the true magnitude of stress and strain applied to the lung both globally and regionally [14]. Moreover, these measurements represent the total elastic pressure of the respiratory system (combining the lung and the chest wall); elevated values therefore do not necessarily entail excess lung stress when chest wall elastance is increased. A rapid and non-invasive technique for detecting excess respiratory effort and dynamic lung stress would substantially increase the feasibility of detecting injurious spontaneous breathing during mechanical ventilation.

During a randomly applied end-expiratory airway occlusion on the ventilator, the airway pressure deflection generated by the patient’s respiratory effort against the occluded airway (Δ*P*_occ_) is correlated with the pressure generated by the respiratory muscles to expand the lungs and chest wall during mechanically assisted breaths because a single end-expiratory occlusion does not alter respiratory drive [15]. Hence, Δ*P*_occ_ may provide a non-invasive means of detecting excessive inspiratory effort and dynamic lung stress during assisted mechanical ventilation.

We hypothesized that excessive patient inspiratory effort (*P*_mus_) and excessive dynamic lung stress (Δ*P*_L,dyn_) could be detected rapidly and non-invasively by measuring Δ*P*_occ_.

## Methods

This study was conducted in two medical-surgical intensive care units at the University Health Network, Toronto, Canada. The findings presented in this paper represent an ancillary analysis on an ongoing clinical study (MYOTRAUMA, ClinicalTrials.gov NCT03108118) characterizing diaphragm activity and function longitudinally during mechanical ventilation. Informed consent was obtained from substitute decision makers prior to enrolment. If no substitute decision maker was available, eligible patients were enrolled by deferred consent and consent for the use of study data was obtained from study participants once they regained capacity. The Research Ethics Board at University Health Network approved the study protocols, and the study was performed in accordance with the ethical standards laid down in the 2008 Declaration of Helsinki. The study findings were validated in a dataset collected from a previously published cohort of patients in China [16].

### Study subjects

Patients were enrolled in the MYOTRAUMA study if they were intubated for fewer than 36 h and if the reason for intubation was one of acute brain injury (i.e., stroke or traumatic brain injury), acute respiratory distress syndrome (ARDS), septic shock, or pneumonia. Patients were excluded if they were deemed unlikely to remain on the ventilator for at least 7 days, if there was a contraindication to esophageal catheterization (recent upper GI surgery, bleeding varices), or if they had a concomitant acute exacerbation of obstructive airways disease. Recordings obtained in MYOTRAUMA study subjects were included from days when the subjects were breathing spontaneously (triggering the ventilator).

### Study protocol

**Fig. 1:**
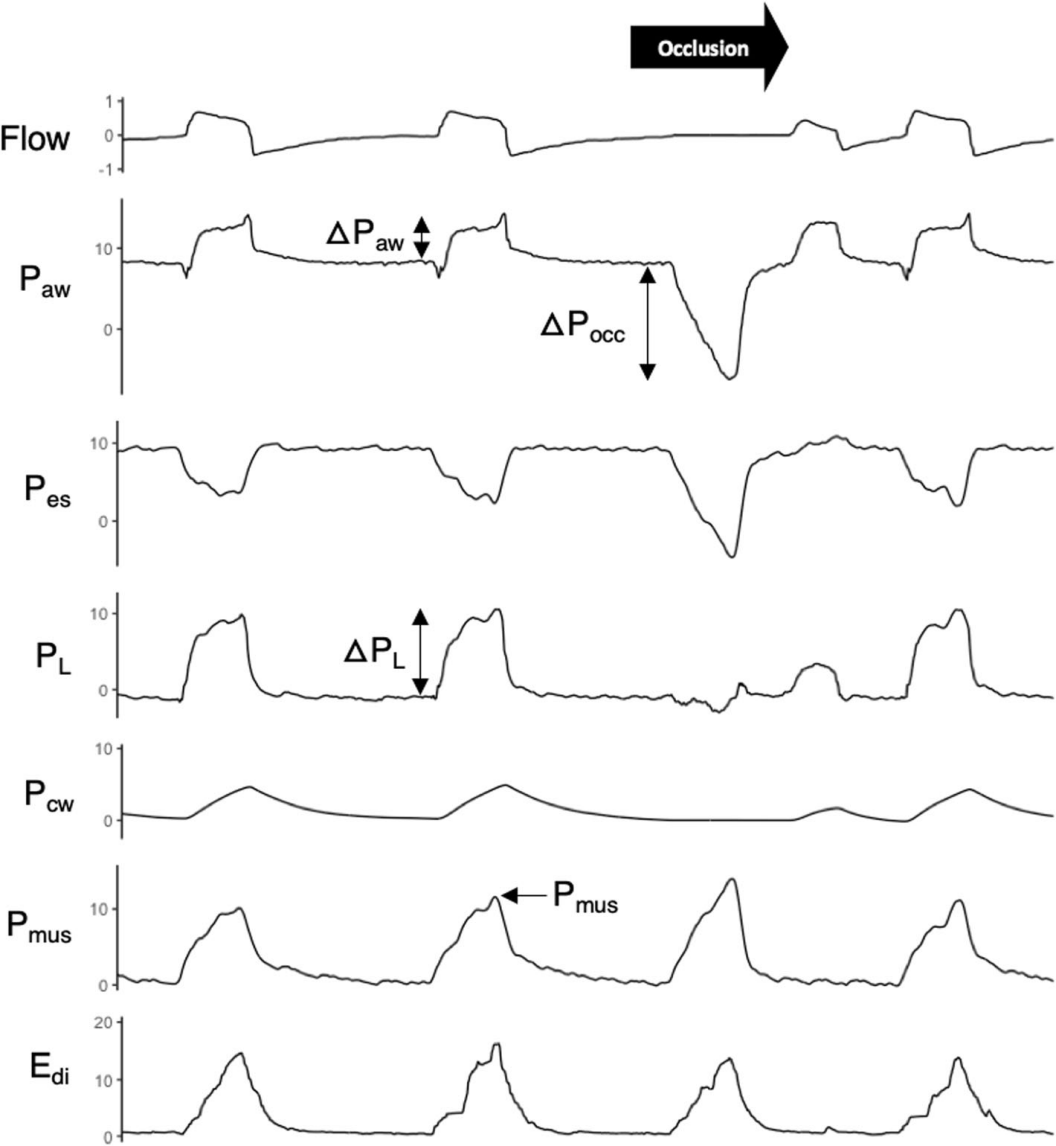
Representative tracings obtained during the airway occlusion maneuver. Flow, airway pressure (*P*_aw_), esophageal pressure (*P*_es_), and diaphragm electrical activity (*E*_di_) were recorded while a one-way end-expiratory occlusion permitting expiratory flow but not inspiratory flow (black arrow) was applied at a random interval. Transpulmonary pressure (P_L_), obtained by digital subtraction of *P*_es_ from *P*_aw_, signifies the dynamic stress applied to the lung. Chest wall elastic recoil pressure (Δ*P*_cw_) was estimated by multiplying tidal volume by predicted chest wall elastance. Inspiratory effort was quantified by the peak inspiratory muscle pressure, *P*_mus_, estimated as the difference between Δ*P*_cw_ and Δ*P*_es_ (baseline *P*_mus_ is 0 cm H_2_O by definition). Note that peak *E*_di_ did not differ between occluded and non-occluded breaths

Study methods are detailed in an online supplement (see Additional file 1). Flow, airway pressure (*P*_aw_), esophageal pressure (*P*_es_), and diaphragm electrical activity (*E*_di_) were recorded for 10 min on a daily basis. During each recording, 15–20 expiratory airway occlusions were applied on the Servo-I ventilator (Getinge, Solna, Sweden) at random intervals. Each occlusion was maintained for the duration of a single breath (confirmed by the return of *P*_aw_ and *E*_di_ to baseline, see Fig. 1). The maximal deflection in *P*_aw_ from PEEP during each occlusion was recorded as a measurement of occlusion pressure (Δ*P*_occ_) (note: not to be confused with the airway occlusion pressure at 100 milliseconds after the onset of inspiration, *P*_0.1_).

### Signal analysis

Transpulmonary pressure (*P*_L_) was measured by real-time digital subtraction of *P*_es_ from *P*_aw_. The airway driving pressure (Δ*P*_aw,dyn_) was quantified as the difference between peak *P*_aw_ and PEEP. The dynamic transpulmonary driving pressure (Δ*P*_L,dyn_) was quantified for each breath as the increase in *P*_L_ from onset to peak during inspiration. Chest wall elastic recoil pressure at end-inspiration (*P*_cw_) was estimated for each breath from the product of tidal volume and the empirically estimated chest wall elastance (see Additional file 1, Additional file 2, Additional file 3, Additional file 4, and Additional file 5). The pressure generated by the respiratory muscles during inspiration (*P*_mus_) (i.e., the pressure that expands the lung and chest wall during inspiration) was quantified for each breath as the peak difference between *P*_cw_ and *P*_es_ during inspiration. Pressure-time product of *P*_mus_ per breath (PTP_mus_)—the reference standard for quantifying inspiratory effort [17]—was computed from *P*_cw_ and the integral of *P*_es_ during inspiration (see Additional file 1, Additional file 2, Additional file 3, Additional file 4, and Additional file 5).

To avoid measurement error due to inaccurate *P*_es_ measurements, recordings where the ratio of Δ*P*_occ_/Δ*P*_es_ was greater than 1.3 or less than 0.7 were excluded from analysis.

### Defining excessive *P*_mus_ and Δ*P*_L_

Thresholds defining excessive *P*_mus_ and Δ*P*_L,dyn_ were selected a priori based on available physiological and clinical observations (see Additional file 1 for detailed rationale). *P*_mus_ normally ranges between 4 and 10 cm H_2_O, and Δ*P*_L,dyn_ normally ranges between 4 and 8 cm H_2_O [18–20]. Given some uncertainty in the optimal definitions for excessive *P*_mus_ and Δ*P*_L,dyn_, discriminative accuracy was assessed for two different possible definitions of “excessive” values: for *P*_mus_, 10 cm H_2_O and 15 cm H_2_O, and for Δ*P*_L,dyn_, 15 cm H_2_O and 20 cm H_2_O.

### Statistical analysis

The goal of the analysis was to determine whether Δ*P*_occ_ measured during airway occlusions could be used to predict the average values of Δ*P*_L,dyn_ and *P*_mus_ for non-occluded (assisted) breaths during each daily 10-min recording and to detect when the average values of Δ*P*_L,dyn_ and *P*_mus_ for non-occluded (assisted breaths) exceeded the cut-off values defined above.

For internal validation, we employed a cross-validation procedure (100 repetitions). During each cross-validation, patients were randomly divided into derivation (*n* = 10, 50%) and internal validation (*n* = 10, 50%) cohorts. In the derivation cohort (step 1), the ratios of mean *P*_mus_ (during all non-occluded breaths) to the mean Δ*P*_occ_ (*k*_1_ = *P*_mus_/Δ*P*_occ_) and mean Δ*P*_es_ (during all non-occluded breaths) to the mean Δ*P*_occ_ (*k*_2_ = Δ*P*_es_/Δ*P*_occ_) were computed in each daily recording using linear mixed-effects models to account for repeated recordings within subjects.

In the internal validation cohort (step 2), the derived values of *k*_1_ and *k*_2_ were used to predict *P*_mus_ and Δ*P*_es_ (and hence Δ*P*_L,dyn_ as Δ*P*_aw_ − Δ*P*_es_) from three randomly selected measurements of Δ*P*_occ_ in each recording (to mimic the use of just three occlusion maneuvers for prediction in clinical practice) according to Eqs. 1 and 2.

1 $$ {P}_{\mathrm{mus},\mathrm{predicted}}={k}_1\times {\Delta P}_{\mathrm{occ}} $$

2 $$ {\Delta P}_{\mathrm{L},\mathrm{dyn},\mathrm{predicted}}={\Delta P}_{\mathrm{aw}}-{k}_2\times {\Delta  P}_{\mathrm{occ}} $$

Predicted and observed values of *P*_mus_ and Δ*P*_L,dyn_ were compared using Bland-Altman limits of agreement. To account for repeated measures within patients, linear mixed-effects models were employed to estimate within-patient limits of agreement as a proportion of the estimated value (LA_%,within_) [21]. Values were log-transformed because of non-normality in the distribution of differences between predicted and estimated values [22]. The mean and between-patient standard deviation of the bias between measured and predicted *P*_mus_ and Δ*P*_L_ (SD_bias,btw_) were also computed in linear mixed-effects models. Total limits of agreement for *P*_mus_ and Δ*P*_L_ across the range of estimated values were estimated as 1.96 × SD_bias,btw_ + LA_%,within_ × estimated value. The ability of predicted *P*_mus_ and Δ*P*_L,dyn_ to detect excessive *P*_mus_ and Δ*P*_L,dyn_ (defined by above threshold values) was evaluated in the internal validation cohort by receiver operating characteristic curve analysis and by computing sensitivity and specificity.

The cross-validation procedure (steps 1 and 2) was repeated 100 times to evaluate the stability of validity estimates during repeated random sampling [23]. All statistical analyses were conducted using R version 3.4.3 (www.r-project.org).

### External validation

The discriminative validity and sensitivity and specificity of predicted *P*_mus_ and Δ*P*_L_ to detect excessive *P*_mus_ and Δ*P*_L,dyn_ were independently quantified in a separate previously published cohort of patients studied in a different center (Nanjing, China) receiving partially assisted ventilation in whom random expiratory airway occlusions were applied at varying levels of ventilator support (*n* = 13) [16].

## Results

### Prevalence of excessive respiratory effort and dynamic lung stress

After excluding 30 recordings because the ratio of Δ*P*_occ_/Δ*P*_es_ was greater than 1.3 or less than 0.7, a total of 52 daily recordings were available in 16 subjects (median 3, IQR 2–5 daily recordings per patient); representative tracings are shown in Fig. 1. Twelve patients were available in the external validation cohort. Patient characteristics in both cohorts are summarized in Table 1.

**Table 1:** Patient characteristics

Patient characteristic	Primary cohort (*n* = 16)	External validation cohort (*n* = 12)
*N* measurements in cohort	52	46
*N* measurements per patient^a^	3 (2–5)	3 (1–7)
Age (years) (mean, SD)	63 (10)	60 (57–73)
Sex (*n*, % female)	7 (44%)	10 (83%)
Cause of respiratory failure (*n*, %)		
Pneumonia	10 (62%)	10 (83%)
Non-pulmonary sepsis	2 (13%)	0 (0%)
Cardiogenic shock	0 (0%)	2 (17%)
Intracranial hemorrhage	3 (19%)	0 (0%)
Ischemic stroke	1 (6%)	0 (0%)
Sedation-Agitation Scale score^b^	2 (2–3)	Not reported
Baseline nadir PaO_2_/FiO_2_ (mm Hg)	148 (105–173)	Not reported
Mode of ventilation (*n* days, %)		
Volume assist-control	1 (2%)	–
Pressure assist-control	9 (17%)	–
Pressure support	39 (75%)	–
Not recorded	3 (6%)	–
Neurally adjusted ventilatory assist	0 (0%)	12 (100%)
Δ*P*_aw_ (cm H_2_O)^b^	5 (3–7)	10 (9–17)
*P*_mus_ (cm H_2_O)^b^	16 (12–22)	7 (5–9)
Δ*P*_L_ (cm H_2_O)^b^	18 (14–23)	18 (14–22)

Results are presented as median and interquartile range unless otherwise reported

^a^In the primary cohort, one measurement was obtained per day; in the external validation cohort, multiple measurements were obtained on the same day at varying NAVA support levels

^b^Values reported include repeated measurements within subjects over different study days

**Fig. 2:**
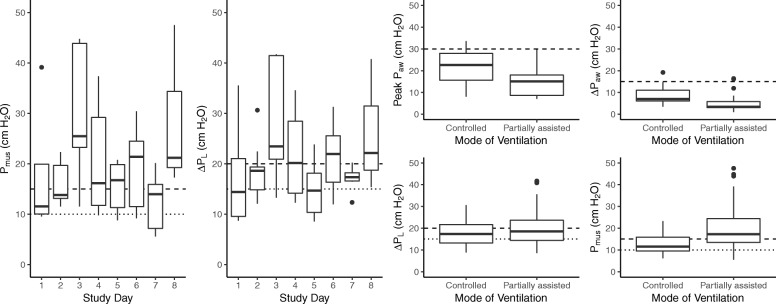
Distribution of Δ*P*_L_ (dynamic transpulmonary driving pressure) and *P*_mus_ (respiratory muscle pressure) during mechanical ventilation. Pressures frequently exceeded “probably excessive” and “definitely excessive” thresholds (dotted and dashed lines, respectively) irrespective of the duration of the study or the mode of ventilation. While peak and driving airway pressures were lower under partially assisted modes of ventilation (*p* < 0.001 for both comparisons), transpulmonary pressure swings were not significantly different (*p* = 0.16)

*P*_mus_ and Δ*P*_L,dyn_ during assisted ventilation ranged widely in the cohort (Fig. 2). *P*_mus_ exceeded 10 cm H_2_O on 84% of patient-days in the study and exceeded 15 cm H_2_O on 53% of patient-days. In 14 patients (88%), *P*_mus_ exceeded 10 cm H_2_O on at least one study day. There was no evidence of a correlation between *P*_mus_ and pH (*p* = 0.21) or PaO_2_ (*p* = 0.57). The correlations between *P*_mus_ and SAS score (*p* = 0.07, *R*^2^ = 0.06) and SOFA score (*p* = 0.08, *R*^2^ = 0.09) did not reach significance. *P*_mus_ was inversely correlated with PaCO_2_ (*p* = 0.03, *R*^2^ = 0.11). *P*_mus_ was higher under partially assisted modes (mean difference 8 cm H_2_O, *p* = 0.02) and higher in patients admitted for pneumonia compared to patients with non-pulmonary admission diagnoses (mean difference 11 cm H_2_O, *p* = 0.05).

Δ*P*_L,dyn_ exceeded 15 cm H_2_O on 69% of patient-days and exceeded 20 cm H_2_O on 40% of patient-days. In 13 patients (81%), Δ*P*_L,dyn_ exceeded 15 cm H_2_O on at least one study day. Δ*P*_L,dyn_ was generally substantially higher than Δ*P*_aw_ because pleural pressure (represented by *P*_es_) decreases during inspiration even while *P*_aw_ increases (median difference 12 cm H_2_O, IQR 8–18 cm H_2_O, *p* < 0.001). Although peak airway pressure and airway driving pressure were lower on days when patients were ventilated in pressure support ventilation mode compared to volume or pressure-control ventilation (*p* < 0.005 for both comparisons), Δ*P*_L,dyn_ was not significantly different (*p* = 0.16) (Fig. 2). Δ*P*_L,dyn_ was higher in patients admitted for pneumonia compared to patients with a non-pulmonary diagnosis (mean difference 9 cm H_2_O, *p* = 0.05).

*P*_mus_ and Δ*P*_L,dyn_ were both within ideal limits (*P*_mus_ ≤ 10 cm H_2_O and Δ*P*_L,dyn_ < 15 cm H_2_O) on only 8% of patient-days.

### Validity of Δ*P*_occ_ as a non-invasive marker of respiratory effort

There was no systematic difference in peak *E*_di_ between occluded and non-occluded breaths (mean difference 0 μV, limits of agreement ± 4 μV) confirming that respiratory drive was unaffected by the randomly applied intermittent airway occlusion. Δ*P*_occ_ was highly correlated with PTP_mus_ (Additional file 2: Figure S1, between-subjects *R*^2^ = 0.71, within-subjects *R*^2^ = 0.85).

### Detecting excessive *P*_mus_ and Δ*P*_L_ from Δ*P*_occ_

In the derivation cohorts, *k*_1_ (ratio of *P*_mus_/Δ*P*_occ_) was − 0.74 (95% CI − 0.69, − 0.78) and *k*_2_ (ratio of Δ*P*_es_/Δ*P*_occ_) was 0.66 (0.61–0.70).

**Fig. 3:**
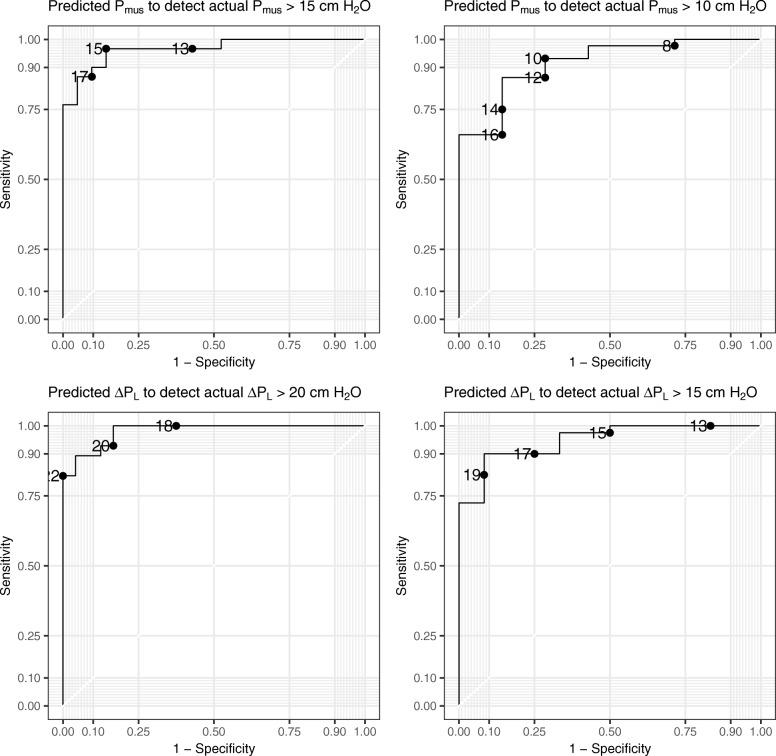
Discriminative accuracy assessed by receiver operating characteristic curves. Threshold values are shown as points on the ROC curves. *P*_mus_, respiratory muscle pressure; Δ*P*_L_, dynamic transpulmonary driving pressure

Agreement between predicted and measured values of *P*_mus_ and Δ*P*_L,dyn_ in the internal validation cohorts was marginally acceptable: bias (the magnitude of difference between predicted and measured values) varied between subjects and the within-subject limits of agreement were relatively wide (Additional file 3: Figure S2, Additional file 4: Table S1**)**. Nevertheless, predicted *P*_mus_ and Δ*P*_L,dyn_ accurately detected excessive *P*_mus_ and Δ*P*_L,dyn_ with areas under the receiver operating characteristic curves (AUROC) suggesting strong discriminative performance (AUROC > 0.9 in all cases, Fig. 3, Additional file 5: Table S2). Sensitivity and specificity of different cut-off values of predicted *P*_mus_ and Δ*P*_L,dyn_ for excessive measured *P*_mus_ and Δ*P*_L,dyn_ are shown in Additional file 5: Table S2.

Based on the findings in the primary cohort, the utility of Δ*P*_occ_ was tested in the external validation cohort using values of *k*_1_ = -3/4 and *k*_2_ = 2/3. Discriminative performance, sensitivity, and specificity for excessive *P*_mus_ and Δ*P*_L,dyn_ were similarly strong (AUROC ≥ 0.94 for both excessive *P*_mus_ and Δ*P*_L,dyn_, Additional file 5: Table S2).

## Discussion

We demonstrate for the first time that measurement of Δ*P*_occ_ from three randomly applied end-expiratory occlusion maneuvers can detect excessive *P*_mus_ and Δ*P*_L,dyn_ with high sensitivity and specificity, even though agreement between predicted and measured values are not sufficiently reliable to provide direct estimates of *P*_mus_ and Δ*P*_L,dyn_. Second, we report for the first time that in spontaneously breathing patients under mechanical ventilation, inspiratory effort and dynamic lung stress frequently exceed putative safe thresholds, irrespective of the depth of sedation or mode of ventilation. Patients only infrequently exhibited the “ideal” combination of lung and diaphragm-protective ventilation parameters (*P*_mus_ ≤ 10 cm H_2_O and Δ*P*_L,dyn_ ≤ 15 cm H_2_O). The magnitude of dynamic lung stress during spontaneous breathing was often seriously underestimated by airway pressures available on the ventilator, confirming that airway pressures on the ventilator are an unreliable marker of dynamic lung stress when patients are spontaneously breathing.

Our method relies on predicting the swing in pleural pressure (quantified by *P*_es_) under dynamic conditions (airway open) from the swing in airway pressure under quasi-static conditions (airway occluded). Under quasi-static conditions, the swing in pleural pressure matches the swing in airway pressure exactly. The swing in pleural pressure is smaller during inspiration than under quasi-static conditions because of the force-velocity relation of muscle and because of differences in chest wall mechanics and thoracoabdominal motion [24, 25]. Despite these sources of heterogeneity, we found that the conversion factors *k*_1_ and *k*_2_ for converting quasi-static conditions to dynamic conditions were fairly stable between patients and over time. These conversion factors provide the physiological basis for predicting *P*_mus_ and Δ*P*_L,dyn_ from Δ*P*_occ_. Of note, a substantial proportion of recordings had to be excluded because Δ*P*_occ_ differed from Δ*P*_es_ during the occlusion maneuver—this highlights the importance of carefully considering esophageal balloon catheter placement when using *P*_es_ for monitoring.

### Inspiratory effort and dynamic lung stress during assisted mechanical ventilation

The transition to partially assisted modes of ventilation is often regarded as a sign of recovery and progress towards liberation from the ventilator. However, important new insights about the potential for lung injury due to excessive inspiratory effort and the associated increase in global and regional lung stress (a phenomenon referred to as patient self-inflicted lung injury, P-SILI [26]) motivate efforts to avoid excessive effort and lung stress.

Our data suggest that greater attention should be paid to the potential risks of excessive inspiratory effort and dynamic lung stress during assisted mechanical ventilation. Observed *P*_mus_ and Δ*P*_L,dyn_ frequently exceeded putative safe levels of inspiratory effort and lung stress. Reliable and feasible clinical monitoring systems are essential to ensure safe and effective ventilation. Although clinicians ordinarily rely on plateau, peak, and driving airway pressures, these parameters can seriously underestimate the true magnitude of lung stress during spontaneous breathing due to the negative pleural pressure generated by the respiratory muscles, which is usually not measured. Clinicians should therefore avoid relying on airway pressure measurements alone to assess the safety of mechanical ventilation in spontaneously breathing patients.

Measurement of Δ*P*_occ_ as described for the first time in this study offers a highly feasible and sensitive means of detecting excessive inspiratory effort and dynamic lung stress. It is important to note that Δ*P*_L,dyn_ can only be predicted if Δ*P*_occ_ < 0 cm H_2_O; when inspiratory effort is absent, the inspiratory swing in Δ*P*_es_ will be positive, and hence, Δ*P*_L,dyn_ will not be correlated to Δ*P*_occ_.

### Limitations

This study has several limitations. First, *P*_mus_ and ΔP_L,dyn_ were estimated from measurements of airway and esophageal pressure. Due to the presence of active inspiratory efforts, we did not perform end-inspiratory holds to obtain quasi-static pressure measurements although some observations suggest that such measurements are feasible in the presence of inspiratory effort provided expiratory muscle activity is minimal [27, 28]. Consequently, Δ*P*_L,dyn_ as measured in this study represents a “dynamic” measure that may overestimate the actual mechanical stress applied to the lung during tidal ventilation. On the other hand, while Δ*P*_L_ at the inspiratory plateau corresponds to the time of maximal alveolar distension in the non-dependent lung, peak Δ*P*_L,dyn_ is reached at the time point when dependent lung—the region most at risk during spontaneous breathing—is maximally distended by vigorous spontaneous efforts [3]. It may therefore be the more clinically relevant marker of dynamic lung stress in this context. Future studies should determine whether dynamic or quasi-static measurements of ΔP_L_ best reflect regional distending pressures.

Second, *P*_mus_ measurements require measurement of elastic chest wall recoil pressure; owing to the absence of recordings of passive ventilation in most subjects, we relied on empirical estimates of chest wall elastance derived from predicted lung volumes. The reliability of this empiric approach is uncertain, but reassuringly, we found that predicted values of chest wall elastic recoil pressure approximated measured values in patients where direct measurements of chest wall elastance were available (as reported in the Additional file 1).

Third, the number of patients in the primary dataset (*n* = 16) is relatively small, possibly limiting the generalizability of the validation findings. The study population is representative of a broad range of ventilated patients with acute hypoxemic respiratory failure. To avoid overfitting the predicted values of *k*_1_ and *k*_2_ to our dataset and to estimate the precision of our estimates of the limits of agreement, we employed a cross-validation technique. Importantly, the approach to detecting excessive *P*_mus_ and Δ*P*_L,dyn_ from Δ*P*_occ_ performed extremely well in the independent external validation cohort from a different country (China). The generalizability of these findings is also supported by the fact that the value of *k*_1_ estimated in this study (median 0.74) corresponds closely to the value estimated by Bellani et al. when they derived the *P*_mus_ − *E*_di_ index (0.66) in an Italian study [29].

### Clinical implications

**Fig. 4:**
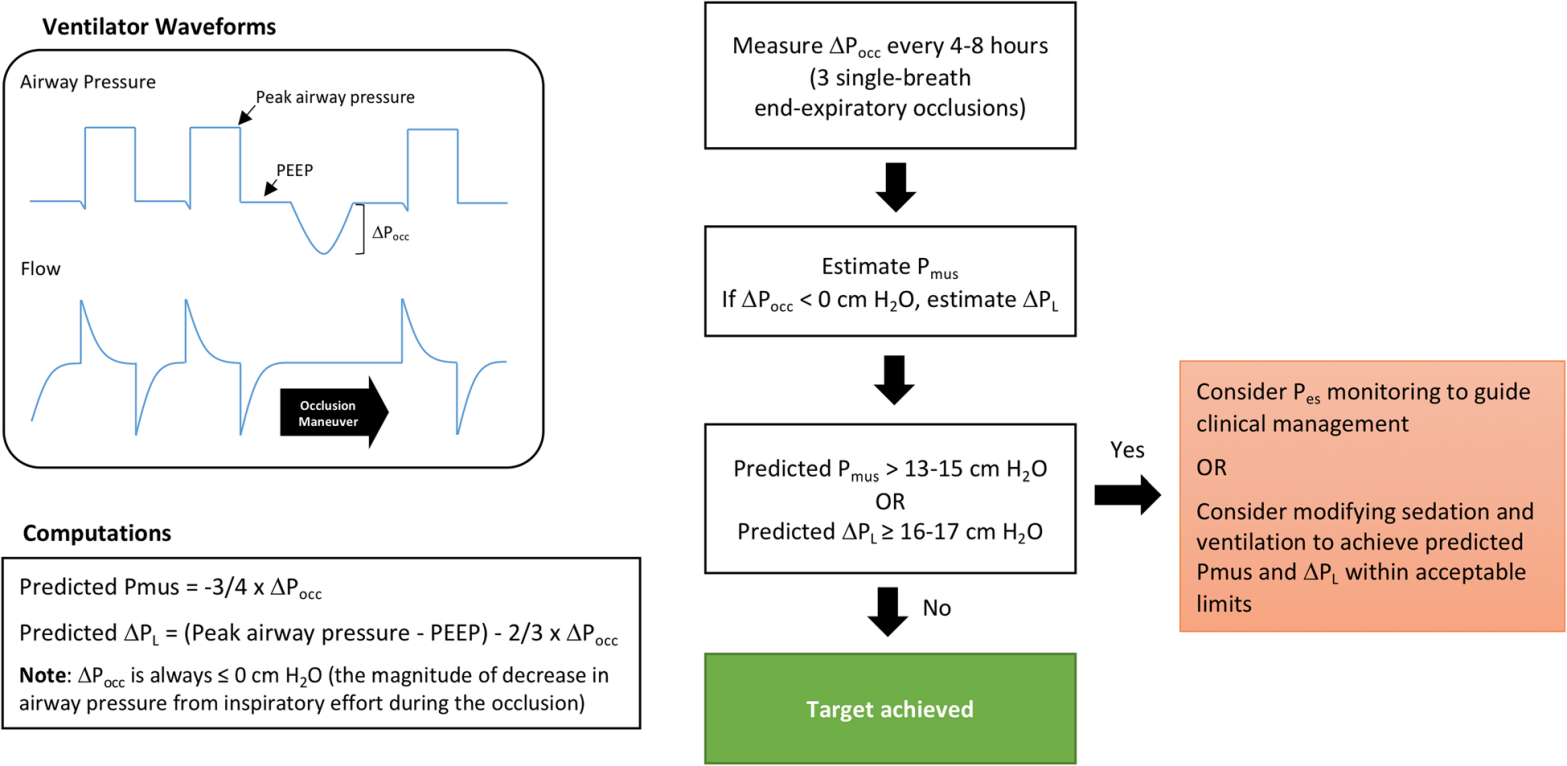
Proposed clinical algorithm for monitoring respiratory muscle pressure (*P*_mus_) and dynamic transpulmonary pressure swings (Δ*P*_L_) based on the negative deflection in airway pressure during an end-expiratory airway occlusion maneuver (Δ*P*_occ_). *P*_es_, esophageal pressure

Regular measurements of Δ*P*_occ_ to estimate *P*_mus_ and Δ*P*_L,dyn_ during mechanical ventilation provide a highly feasible means of detecting excessive respiratory effort and excessive dynamic lung stress directly from ventilator waveforms. Most modern ventilators have capacity to apply an end-expiratory occlusion during ventilation in controlled or partially assisted modes. *P*_mus_ and Δ*P*_L,dyn_ values predicted from Δ*P*_occ_ are not sufficiently accurate to replace direct clinical monitoring (i.e., esophageal pressure) if desired by clinicians. Rather, these estimates could be used as a highly feasible, rapid, non-invasive “screening test” for excessive *P*_mus_ and Δ*P*_L,dyn_. These data could be employed as an indication to deploy more direct monitoring techniques (i.e., esophageal manometry) or to guide adjustments to ventilator assist level, sedation, and opioids (Fig. 4). The maneuver was well-tolerated in our study.

## Conclusions

Inspiratory effort and dynamic lung stress often exceed safe limits in patients breathing spontaneously under mechanical ventilation. The airway pressure deflection resulting from patient inspiratory effort during a transient end-expiratory occlusion maneuver (Δ*P*_occ_) can be used to detect excessive (potentially injurious) inspiratory effort and dynamic lung stress.

## Supplementary information

**Additional file 1.** Supplemental description of methods.

**Additional file 2: Figure S1.** ΔPocc is correlated with inspiratory effort quantified by the pressure-time product of Pmus.

**Additional file 3: Figure S2.** Accuracy of predicting Pmus and ΔP_L_ from ΔPocc assessed by Bland-Altman plots.

**Additional file 4: Table S1.** Agreement between measured and predicted Pmus and ΔP_L_.

**Additional file 5: Table S2.** Discriminative performance of predicted Pmus and ΔP_L_ values to detect excessive Pmus and ΔP_L_.

## Data Availability

The datasets generated and/or analyzed during the current study are not publicly available due to ongoing analysis in the primary study but are available from the corresponding author on reasonable request.

## Abbreviations

Δ*P*_aw,dyn_: Inspiratory swing in pressure (difference between peak *P*_aw_ and PEEP)
Δ*P*_L,dyn_: Dynamic transpulmonary driving pressure; increase in P_L_ from onset to peak during inspiration
Δ*P*_L,dyn_: Dynamic transpulmonary driving pressure swing from end-expiration to peak transpulmonary pressure
Δ*P*_occ_: Airway pressure deflection generated by the patient’s respiratory effort against the occluded airway; airway pressure from PEEP. The time of occlusion is as long as the duration of a single breath
*E*_di_: Diaphragm electrical activity
*k*_1_: The ratio of mean *P*_mus_ (during all non-occluded breaths) to the mean Δ*P*_occ_ (*k*_1_ = *P*_mus_/Δ*P*_occ_)
*k*_2_: The ratio of mean Δ*P*_es_ (during all non-occluded breaths) to the mean Δ*P*_occ_ (*k*_2_ = Δ*P*_es_/Δ*P*_occ_)
*P*_0.1_: The airway occlusion pressure at 100 milliseconds after the onset of inspiration
*P*_aw_: Airway pressure
*P*_cw_: Chest wall elastic recoil pressure at end-inspiration
*P*_es_: Esophageal pressure
*P*_L_: Transpulmonary pressure
*P*_mus_: Respiratory muscle pressure **(**inspiratory effort); peak difference between *P*_cw_ and *P*_es_ during inspiration
PTP_mus_: Pressure-time product of *P*_mus_ per breath
SAS score: Sedation-Agitation Scale score
SOFA score: Sequential Organ Failure Assessment score

## References

[1] Yoshida T, Nakahashi S, Nakamura M, Koyama Y, Roldan R, Torsani V (2017). Volume-controlled ventilation does not prevent injurious inflation during spontaneous effort. Am J Resp Crit Care.

[2] Papazian L, Forel J-M, Gacouin A, Penot-Ragon C, Perrin G, Loundou A (2010). Neuromuscular blockers in early acute respiratory distress syndrome. New Engl J Medicine..

[3] Yoshida T, Amato MB, Kavanagh BP (2018). Understanding spontaneous vs ventilator breaths: impact and monitoring. Intens Care Med.

[4] Orozco-Levi M, Lloreta J, Minguella J, Serrano S, Broquetas J, Gea J (2001). Injury of the human diaphragm associated with exertion and chronic obstructive pulmonary disease. Am J Resp Crit Care.

[5] Hussain S, Roussos C (1985). Distribution of respiratory muscle and organ blood flow during endotoxic shock in dogs. J Appl Physiol.

[6] Goligher EC, Dres M, Fan E, Rubenfeld GD, Scales DC, Herridge MS (2018). Mechanical ventilation-induced diaphragm atrophy strongly impacts clinical outcomes. Am J Resp Crit Care.

[7] Vaporidi K, Akoumianaki E, Telias I, Goligher EC, Brochard L, Georgopoulos D. Respiratory drive in critically ill patients: pathophysiology and clinical implications. Am J Respir Crit Care Med 2019;0. 10.1164/rccm.201903-0596SO 31437406

[8] Gentzler Eliza R., Derry Heather, Ouyang Daniel J., Lief Lindsay, Berlin David A., Xu Cici Jiehui, Maciejewski Paul K., Prigerson Holly G. (2019). Underdetection and Undertreatment of Dyspnea in Critically Ill Patients. American Journal of Respiratory and Critical Care Medicine.

[9] Mauri T, Yoshida T, Bellani G, Goligher EC, Carteaux G, Rittayamai N (2016). Esophageal and transpulmonary pressure in the clinical setting: meaning, usefulness and perspectives. Intens Care Med.

[10] Beck J, Gottfried SB, Navalesi P, Skrobik Y, Comtois N, ROSSINI M (2001). Electrical activity of the diaphragm during pressure support ventilation in acute respiratory failure. Am J Resp Crit Care.

[11] Goligher EC, Laghi F, Detsky ME, Farias P, Murray A, Brace D (2015). Measuring diaphragm thickness with ultrasound in mechanically ventilated patients: feasibility, reproducibility and validity. Intens Care Med.

[12] Telias I, Damiani F, Brochard L (2018). The airway occlusion pressure (P0.1) to monitor respiratory drive during mechanical ventilation: increasing awareness of a not-so-new problem. Intens Care Med.

[13] Amato MB, Meade MO, Slutsky AS, Brochard L, Costa EL, Schoenfeld DA (2015). Driving pressure and survival in the acute respiratory distress syndrome. New Engl J Med.

[14] Bellani G, Grasselli G, Teggia-Droghi M, Mauri T, Coppadoro A, Brochard L (2016). Do spontaneous and mechanical breathing have similar effects on average transpulmonary and alveolar pressure? A clinical crossover study. Crit Care.

[15] Xirouhaki N, Kondili E, Mitrouska I, Siafakas N, Georgopoulos D (1999). Response of respiratory motor output to varying pressure in mechanically ventilated patients. Eur Respiratory J.

[16] Liu L, Liu S, Xie J, Yang Y, Slutsky AS, Beck J (2015). Assessment of patient-ventilator breath contribution during neurally adjusted ventilatory assist in patients with acute respiratory failure. Crit Care.

[17] Jubran A, Tobin M (1997). Pathophysiologic basis of acute respiratory distress in patients who fail a trial of weaning from mechanical ventilation. Am J Resp Crit Care.

[18] Carteaux G, Mancebo J, Mercat A, Dellamonica J, Richard J-CM, Aguirre-Bermeo H (2013). Bedside adjustment of proportional assist ventilation to target a predefined range of respiratory effort. Crit Care Med.

[19] Mancebo J, Isabey D, Lorino H, Lofaso F, Lemaire F, Brochard L (1995). Comparative effects of pressure support ventilation and intermittent positive pressure breathing (IPPB) in non-intubated healthy subjects. Eur Respir J.

[20] Jubran Amal, Grant Brydon J. B., Laghi Franco, Parthasarathy Sairam, Tobin Martin J. (2005). Weaning Prediction. American Journal of Respiratory and Critical Care Medicine.

[21] Myles P, CUI J (2007). Using the Bland-Altman method to measure agreement with repeated measures. Bja Br J Anaesth.

[22] Euser AM, Dekker FW, le Cessie S (2008). A practical approach to Bland-Altman plots and variation coefficients for log transformed variables. J Clin Epidemiol.

[23] Altman DG, Royston P (2000). What do we mean by validating a prognostic model?. Stat Med.

[24] Grassino A, Goldman, Mead J, Sears T (1978). Mechanics of the human diaphragm during voluntary contraction: statics. J Appl Physiol.

[25] Goldman GA, Mead J, Sears T (1978). Mechanics of the human diaphragm during voluntary contraction: dynamics. J Appl Physiol.

[26] Brochard L, Slutsky A, Pesenti A (2017). Mechanical ventilation to minimize progression of lung injury in acute respiratory failure. Am J Resp Crit Care.

[27] Bellani G, Grassi A, Sosio S, Gatti S, Kavanagh BP, Pesenti A (2019). Driving pressure is associated with outcome during assisted ventilation in acute respiratory distress syndrome. Anesthesiology..

[28] Bellani G, Grassi A, Sosio S, Foti G (2019). Plateau and driving pressure in the presence of spontaneous breathing. Intens Care Med.

[29] Bellani G, Mauri T, Coppadoro A, Grasselli G, Patroniti N, Spadaro S (2013). Estimation of patient’s inspiratory effort from the electrical activity of the diaphragm. Crit Care Med.

