# Supplementary material for: A novel non-invasive method to detect excessively high respiratory effort and dynamic transpulmonary driving pressure during mechanical ventilation
Source: Crit Care. 2019 Nov 6;23:346. doi: 10.1186/s13054-019-2617-0 (PMC6836358; doi:10.1186/s13054-019-2617-0)
Supplement: Supplementary file 1 — Additional file 1. Supplemental description of methods. [file 13054_2019_2617_MOESM1_ESM.docx]

Online Supplement

**A Novel Non-Invasive Method to Detect Excessively High Respiratory Effort and Transpulmonary Driving Pressure During Mechanical Ventilation**

Michele Bertoni et al.

**Supplemental Description of Study Methods**

*Study Measurements*

Upon enrollment in MYOTRAUMA, a nasogastric catheter fitted with esophageal and gastric balloons and a multiple electrode array for diaphragm electromyography (EMG) was placed. EMG signals were acquired, filtered and processed to obtain the diaphragm EMG root mean square (diaphragm electrical activity, Edi) by the Servo-i mechanical ventilator (Maquet, Solna, Sweden) according to previously published methods [1]. Airway pressure (Paw), flow, and Edi were recorded in real time at a sampling frequency of 62.5 Hz by a personal computer connected to the ventilator using dedicated software (Neurovent Inc., Toronto, Canada). Esophageal and gastric balloons were coupled to pressure transducers attached to the Neurovent ® monitoring system. Catheter positioning was validated on a daily basis based on the electrode signals and by the Baydur maneuver [2].

*Study Protocol*

Patient inspiratory effort during ventilation was monitored continuously for up to 7 days (or until extubation or death, if earlier). On days when patient inspiratory effort was present (i.e. the patient was triggering the ventilator), 15-20 end-expiratory airway occlusions lasting for the duration of a single inspiratory cycle (i.e. until Edi returned to baseline, approximately 1-2 seconds) were applied intermittently using the expiratory occlusion valve on the Servo-i ventilator over a 10-minute period. Flow, Paw, Pes, and Edi were recorded during this 10-minute period. Representative tracings of pressure and flow during the airway occlusion maneuver are displayed in Figure 1. No adjustments were made to ventilation or sedation prior to or during the measurement session.

*Signal Analysis*

Inspirtory swings in Paw and Pes from baseline to peak were computed as ΔP_aw,dyn_ and ΔPes, respectively. The peak value of Edi was recorded as Edi. Dynamic transpulmonary driving pressure (ΔP_L,dyn_) was computed as the difference between ΔPaw and ΔPes.

where Ti represents inspiratory time (i.e. time T from end-expiration to end-inspiration).

Peak inspiratory muscle pressure (Pmus) was calculated as the difference between the peak inspiratory chest wall elastic recoil pressure (Pcw) and ΔPes; Pcw was computed as the product of tidal volume and chest wall elastance (Ecw) [2]. We empirically estimated Ecw for all patients based on predicted vital capacity (3, 4). In 8 patients in whom direct measurements of chest wall elastance were available, predicted and measured Pcw were similar (mean 4.1 cm H_2_O, bias 0.4 cm H_2_O, limits of agreement -1.2 to 2.0 cm H_2_O) confirming that empirically estimated Pmus was acceptably accurate.

To correct Pmus for abdominal muscle relaxation, the expiratory rise in Pga (if present) was subtracted from Pmus [3]. The pressure-time product of Pmus per breath (PTPmus) was estimated as the sum of the pressure-time product of Pes (integral of Pes from baseline to end-inspiration) and the pressure-time product of Pcw (estimated as the product of peak Pcw and inspiratory time multiplied by 0.5) [4] as per Equation 1.

$PTP_{mus}=\frac{Pcw\cdot Ti}{2}+\int_{end-expiration}^{end-inspiration} Pes\cdot dT$(Equation 1)

*Defining Excessive Pmus and ΔP_L,dyn_*

We selected threshold values for excessive Pmus and ΔP_L,dyn_ on an *a priori* basis given several physiological and clinical considerations:

- First, during resting quiet breathing and during successful weaning trials, Pmus ranges between approximately 4 to 10 cm H_2_O and ΔP_L,dyn_ ranges between 4-8 cm H_2_O [4-6]. This circumstantially sorts this range of values as a level of inspiratory effort that is safe and generally well-tolerated over long periods of time.
- Second, in mechanically ventilated patients, maximal inspiratory pressure is often markedly reduced (in the range of 30-40 cm H_2_O) [7,8] such that Pmus levels exceeding 10-15 cm H_2_O are likely to reach a value of respiratory muscle tension-time index consistent with fatigue or injury (assuming an I:E ratio of 1:3 or lower) [9,10].
- Third, recent observations suggesting that diaphragm thickening fraction levels above 30% (near the upper limit of values observed during resting breathing) are associated with increases in diaphragm thickness and prolonged mechanical ventilation [11].
- Fourth, because the specific elastance of unaffected (‘baby’) lung is approximately 13 cm H_2_O, ΔP_L_ (a measure of lung stress) in the range of 15 to 20 cm H_2_O reflects lung strain values of approximately 1.2 to 1.5 [12]—limits of lung strain tolerated in experimental studies of lung injury [13,14]. Some data suggest that ΔP_L,dyn_ above 20 cm H_2_O are associated with a high risk of death in patients with severe ARDS [15]; other data suggest even more conservative thresholds for excessive lung stress (≥10 cm H_2_O) [16].

**References**

1. Sinderby C, Navalesi P, Beck J, Skrobik Y, Comtois N, Friberg S, et al. Neural control of mechanical ventilation in respiratory failure. Nat. Med. 1999;5:1433–6.

2. Mauri T, Yoshida T, Bellani G, Goligher EC, Carteaux G, Rittayamai N, et al. Esophageal and transpulmonary pressure in the clinical setting: meaning, usefulness and perspectives. Intensive Care Medicine. 2016;42:1360–73.

3. Lessard MR, Lofaso F, Brochard L. Expiratory muscle activity increases intrinsic positive end-expiratory pressure independently of dynamic hyperinflation in mechanically ventilated patients. Am. J. Respir. Crit. Care Med. 1995;151:562–9.

4. Carteaux G, Mancebo J, Mercat A, Dellamonica J, Richard J-CM, Aguirre-Bermeo H, et al. Bedside adjustment of proportional assist ventilation to target a predefined range of respiratory effort. Critical Care Medicine. 2013;41:2125–32.

5. Mancebo J, Isabey D, Lorino H, Lofaso F, Lemaire F, Brochard L. Comparative effects of pressure support ventilation and intermittent positive pressure breathing (IPPB) in non-intubated healthy subjects. Eur Respir J. 1995;8:1901–9.

6. Jubran A, Grant BJB, Laghi F, Parthasarathy S, Tobin MJ. Weaning prediction: esophageal pressure monitoring complements readiness testing. Am. J. Respir. Crit. Care Med. 2005;171:1252–9.

7. Supinski GS, Westgate P, Callahan LA. Correlation of maximal inspiratory pressure to transdiaphragmatic twitch pressure in intensive care unit patients. Critical care (London, England). 2016;20:77.

8. Caruso P, Carnieli DS, Kagohara KH, Anciães A, Segarra JS, Deheinzelin D. Trend of maximal inspiratory pressure in mechanically ventilated patients: predictors. Clinics. 2008;63:33–8.

9. Bellemare F, Grassino A. Effect of pressure and timing of contraction on human diaphragm fatigue. 1982;53:1190–5.

10. Laghi F, D'Alfonso N, Tobin MJ. Pattern of recovery from diaphragmatic fatigue over 24 hours. J Appl Physiol. 1995;79:539–46.

11. Goligher EC, Dres M, Fan E, Rubenfeld GD, Scales DC, Herridge MS, et al. Mechanical Ventilation-induced Diaphragm Atrophy Strongly Impacts Clinical Outcomes. Am. J. Respir. Crit. Care Med. 2018;197:204–13.

12. Chiumello D, Carlesso E, Cadringher P, Caironi P, Valenza F, Polli F, et al. Lung stress and strain during mechanical ventilation for acute respiratory distress syndrome. Am. J. Respir. Crit. Care Med. 2008;178:346–55.

13. Protti A, Andreis DT, Monti M, Santini A, Sparacino CC, Langer T, et al. Lung stress and strain during mechanical ventilation: any difference between statics and dynamics? Critical Care Medicine. 2013;41:1046–55.

14. Protti A, Cressoni M, Santini A, Langer T, Mietto C, Febres D, et al. Lung stress and strain during mechanical ventilation: any safe threshold? Am. J. Respir. Crit. Care Med. 2011;183:1354–62.

15. Chiu L-C, Hu H-C, Hung C-Y, Chang C-H, Tsai F-C, Yang C-T, et al. Dynamic driving pressure associated mortality in acute respiratory distress syndrome with extracorporeal membrane oxygenation. Ann Intensive Care. 3rd ed. 2017;7:12.

16. Baedorf Kassis E, Loring SH, Talmor D. Mortality and pulmonary mechanics in relation to respiratory system and transpulmonary driving pressures in ARDS. Intensive Care Medicine. Springer Berlin Heidelberg; 2016;42:1206–13.

**Supplemental Digital Content Figure Legends**

**Figure E1.** ΔPocc is correlated with inspiratory effort quantified by the pressure-time product of Pmus (between patients R^2^=0.67, within patients R^2^=0.83).

**Figure E2. Accuracy of predicting Pmus and ΔP_L_ from ΔPocc assessed by Bland-Altman plots.**
