# Supplementary material for: A novel non-invasive method to detect excessively high respiratory effort and dynamic transpulmonary driving pressure during mechanical ventilation
Source: Crit Care. 2019 Nov 6;23:346. doi: 10.1186/s13054-019-2617-0 (PMC6836358; doi:10.1186/s13054-019-2617-0)
Supplement: Supplementary file 4 — Additional file 4: Table S1. Agreement between measured and predicted Pmus and ΔPL. [file 13054_2019_2617_MOESM4_ESM.docx]

**Table E1.** Agreement between measured and predicted Pmus and ΔP_L_

|  | **Measurement** | **Estimate**  **(95% confidence intervals)** |
| --- | --- | --- |
| Predicted vs. measured P_mus_ | Mean bias across all patients | 0.1 (-0.1, 0.4) cm H_2_O |
|  | Standard deviation of bias between patients | 2.1 (0.2, 5.2) cm H_2_O |
|  | Limits of agreement within patients  (% of estimated value) | 36% (29%, 46%) |
| Predicted vs. measured ΔP_L_ | Mean bias across all patients | 0.0 (-0.2, 0.1) cm H_2_O |
|  | Standard deviation of bias between patients | 0.3 (0.0, 4.3) cm H_2_O |
|  | Limits of agreement within patients  (% of estimated value) | 28% (21%, 32%) |
