# Supplementary material for: A novel non-invasive method to detect excessively high respiratory effort and dynamic transpulmonary driving pressure during mechanical ventilation
Source: Crit Care. 2019 Nov 6;23:346. doi: 10.1186/s13054-019-2617-0 (PMC6836358; doi:10.1186/s13054-019-2617-0)
Supplement: Supplementary file 5 — Additional file 5: Table S2. Discriminative performance of predicted Pmus and ΔPL values to detect excessive Pmus and ΔPL. [file 13054_2019_2617_MOESM5_ESM.docx]

**Table E2.** Discriminative performance of predicted Pmus and ΔP_L_ values to detect excessive Pmus and ΔP_L_

| **Parameter** | **Operating Definition of Abnormal Condition** | **Area under receiver operating characteristic curve (95% CI)** | | **Cut-off of Predicted Value for Diagnosis of Abnormal Condition** | **Internal Cross-Validation** | | **External Validation** | |
| --- | --- | --- | --- | --- | --- | --- | --- | --- |
|  |  | **Internal Cross-Validation** | **External Validation** |  | **Sensitivity (95% CI)** | **Specificity (95% CI)** | **Sensitivity** | **Specificity** |
| Excessive inspiratory effort | Pmus > 10 cm H_2_O | 0.92 (0.83, 0.97) | 0.96 | Predicted Pmus > 8 cm H_2_O | 96% (91%, 100%) | 33% (00%, 79%) | 100% | 65% |
|  |  |  |  | Predicted Pmus > 9 cm H_2_O | 95% (87%, 100%) | 50% (5%, 100%) | 89% | 84% |
|  |  |  |  | Predicted Pmus > 10 cm H_2_O | 92% (82%, 100%) | 67% (26%, 100%) | 89% | 95% |
|  | Pmus > 15 cm H_2_O | 0.96 (0.91, 0.99) | Not tested* | Predicted Pmus > 13 cm H_2_O | 94% (87%, 100%) | 67% (46%, 86%) | Not tested* | |
|  |  |  |  | Predicted Pmus > 14 cm H_2_O | 93% (84%, 100%) | 79% (53%, 92%) |  |  |
|  |  |  |  | Predicted Pmus > 15 cm H_2_O | 91% (77%, 100%) | 85% (63%, 100%) |  |  |
| Excessive dynamic lung stress | ΔP_L_ > 15 cm H_2_O | 0.93 (0.86, 0.99) | 0.94 | Predicted ΔP_L_ > 15 cm H_2_O | 96% (89%, 100%) | 50% (25%, 77%) | 100% | 36% |
|  |  |  |  | Predicted ΔP_L_ > 16 cm H_2_O | 93% (83%, 100%) | 67% (38%, 100%) | 94% | 71% |
|  |  |  |  | Predicted ΔP_L_ > 17 cm H_2_O | 89% (78%, 99%) | 80% (55%, 100%) | 88% | 93% |
|  | ΔP_L_ > 20 cm H_2_O | 0.97 (0.92, 0.99) | Not tested* | Predicted ΔP_L_ > 18 cm H_2_O | 100% (92%, 100%) | 67% (46%, 87%) | Not tested* | |
|  |  |  |  | Predicted ΔP_L_ > 19 cm H_2_O | 100% (88%, 100%) | 75% (60%, 93%) |  |  |
|  |  |  |  | Predicted ΔP_L_ > 20 cm H_2_O | 93% (78%, 100%) | 83% (69%, 100%) |  |  |

*Not tested owing to insufficient number of measurements satisfying definition of abnormal condition in dataset
